# A phase I dose-finding study of a combination of pegylated liposomal doxorubicin (Doxil), carboplatin and paclitaxel in ovarian cancer

**DOI:** 10.1038/sj.bjc.6600250

**Published:** 2002-05-06

**Authors:** D D Gibbs, L Pyle, M Allen, M Vaughan, A Webb, S R D Johnston, M E Gore

**Affiliations:** Department of Medicine, The Royal Marsden Hospital, Fulham Road, London SW3 6JJ, UK

**Keywords:** ovarian cancer, liposomal doxorubicin, carboplatin, paclitaxel

## Abstract

Standard chemotherapy for advanced epithelial ovarian cancer is a combination of platinum-paclitaxel. One strategy to improve the outcome for patients is to add other agents to standard therapy. Doxil is active in relapsed disease and has a response rate of 25% in platinum-resistant relapsed disease. A dose finding study of doxil-carboplatin-paclitaxel was therefore undertaken in women receiving first-line therapy. Thirty-one women with epithelial ovarian cancer or mixed Mullerian tumours of the ovary were enrolled. The doses of carboplatin, paclitaxel and doxil were as follows: carboplatin AUC 5 and 6; paclitaxel, 135 and 175 mg m^−2^; doxil 20, 30, 40 and 50 mg m^−2^. Schedules examined included treatment cycles of 21 and 28 days, and an alternating schedule of carboplatin-paclitaxel (q 21) with doxil being administered every other course (q 42). The dose-limiting toxicities were found to be neutropenia, stomatitis and palmar plantar syndrome and the maximum tolerated dose was defined as; carboplatin AUC 5, paclitaxel 175 mg m^−2^ and doxil 30 mg m^−2^ q 21. Reducing the paclitaxel dose to 135 mg m^−2^ did not allow the doxil dose to be increased. Delivering doxil on alternate cycles at doses of 40 and 50 mg m^−2^ also resulted in dose-limiting toxicities. The recommended doses for phase II/III trials are carboplatin AUC 6, paclitaxel 175 mg m^−2^, doxil 30 mg m^−2^ q 28 or carboplatin AUC 5, paclitaxel 175 mg m^−2^, doxil 20 mg m^−2^ q 21. Grade 3/4 haematologic toxicity was common at the recommended phase II doses but was short lived and not clinically important and non-haematologic toxicities were generally mild and consisted of nausea, paraesthesiae, stomatitis and palmar plantar syndrome.

*British Journal of Cancer* (2002) **86**, 1379–1384. DOI: 10.1038/sj/bjc/6600250 www.bjcancer.com

© 2002 Cancer Research UK

## 

Epithelial ovarian cancer (EOC) is the fifth commonest cancer in women and despite the use of platinum-based chemotherapy, the prognosis for women with advanced disease remains poor with a 5-year disease-specific survival of 28% (Cancer Research Campaign, 1991). The standard first-line chemotherapy for advanced disease is a platinum-paclitaxel combination. McGuire *et al* (1996) demonstrated that treatment of epithelial ovarian cancer with cisplatin-paclitaxel resulted in improved overall survival compared to treatment with cisplatin-cyclophosphamide. This result was confirmed by the Intergroup study (Piccart *et al*, 2000) and subsequent trials have demonstrated the improved toxicity profile and equal efficacy of carboplatin-paclitaxel compared with cisplatin-paclitaxel (du Bois *et al*, 1999; Neijt and du Bois, 1999; Ozols *et al*, 1999).

It is thought that the poor outcome of ovarian cancer is due to the outgrowth of platinum and paclitaxel resistant clones. Over the last decade, a number of agents have been identified that have activity in platinum and paclitaxel refractory ovarian cancer. The addition of these agents to first-line platinum-paclitaxel regimens, either in combination or sequentially, has been proposed as means of improving results of chemotherapy in this disease.

Doxorubicin has single-agent activity in relapsed ovarian cancer (Hubbard *et al*, 1978) and two meta-analyses of trials using platinum-based therapy (The Ovarian Cancer Meta-Analysis Project, 1991; A'Hern and Gore, 1995) suggest that the addition of anthracyclines increases overall survival. ICON2, a randomised trial comparing single-agent carboplatin with cisplatin-doxorubicin-cyclophosphamide (CAP) did not confirm this suggestion and CAP resulted in greater toxicity without an improvement in outcome (The Icon Collaborators, 1998). There are two trials comparing carboplatin-paclitaxel with carboplatin-paclitaxel-epirubicin as first-line treatment of epithelial ovarian cancer but one is still accruing (EORTC) and the other is not fully reported yet (AGO). Phase I/II trials of doxorubicin combined with carboplatin-paclitaxel on a 3 weekly schedule with G-CSF support or doxorubicin-carboplatin with weekly paclitaxel (Hill *et al*, 1997) show that both regimens are active but have significant toxicity and are only suitable for fit patients.

The liposomal formulation of doxorubicin known as Doxil (or Caelyx) is a formulation of standard doxorubicin encapsulated in pegylated liposomes. Its toxicity profile is different from that of standard doxorubicin and in single-agent trials, the dose-limiting toxicities are stomatitis, myelosuppression and palmar-plantar syndrome (PPS), similar to that seen with prolonged infusional fluorouracil. PPS is said to occur at dose rates of greater than 10 mg m^−2^ per week. Cardiac toxicity is not apparent with cumulative doses exceeding 500 mg m^−2^ (Safra *et al*, 2000).

Doxil has been shown to have single agent activity against relapsed ovarian cancer in a number of trials (Muggia *et al*, 1997; Gordon *et al*, 2000). In the first phase II trial of doxil in 35 women with platinum and paclitaxel-resistant epithelial ovarian cancer, a response rate of 25% and a progression-free survival of 5.7 months was observed, at dose of 50 mg m^−2^ given 4 weekly (Muggia *et al*, 1997). The non-overlapping toxicity profiles and evidence of some degree of non-cross resistance makes the combination of doxil with carboplatin-paclitaxel an attractive prospect.

We therefore undertook a dose-finding study of the combination of doxil, carboplatin and paclitaxel.

## PATIENTS AND METHODS

### Inclusion and exclusion criteria

Patients with a diagnosis of epithelial ovarian cancer, fallopian tube carcinoma, mixed Müllerian tumour or primary peritoneal carcinoma requiring first-line chemotherapy (FIGO stage IC to IV) were eligible for entry into the study. Patients were required to have an ECOG performance status of 0–1, creatinine clearance >60 ml min^−1^, left ventricular ejection fraction ⩾50%, bilirubin and transaminases <twice upper limit normal and adequate haematological function, defined by haemoglobin >10 g dl^−1^, neutrophil count >3×10^9^ l^−1^ and platelets >100×10^9^ l^−1^. Women with a prior history of malignancy were included provided they had a disease-free interval of at least 3 years. Patients with a history of cardiac disease, symptomatic peripheral neuropathy or tumours of borderline histology were not eligible. The trial protocol was approved by the Research Ethics Committee of the Royal Marsden Hospital and all patients were required to give fully informed written consent.

### Treatment

Patients received doxil, followed by carboplatin then paclitaxel. Paclitaxel 135 mg m^−2^ or 175 mg m^−2^ was administered in 5% dextrose, as a 3 h infusion. The dose of carboplatin was calculated according to the Calvert formula (Calvert *et al*, 1989) to achieve an area under the concentration-time curve (AUC) of 5–6 mg ml^−1^ min^−1^. The glomerular filtration rate was estimated by ^51^Cr EDTA clearance.

Carboplatin was given as an i.v. infusion over 1 h and liposomal doxorubicin was administered as a 1-h infusion in 250 ml of 5% dextrose. All patients received the following premedication: dexamethasone 20 mg p.o. 12 and 6 h prior to chemotherapy, chlorpheniramine 10 mg i.v. and cimetidine 300 mg i.v. 30 min prior to chemotherapy and ondansetron 8 mg i.v. with chemotherapy. In addition, patients received dexamethasone 4 mg t.d.s. and metoclopramide 20 mg tds for 4 days after treatment.

The dose escalation schedule is shown in Table 1

**Table 1:**
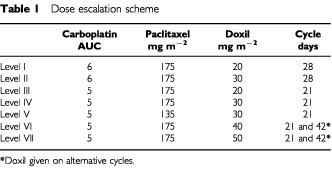
Dose escalation scheme

. The first patient at each dose level was evaluated for toxicity over one full cycle before subsequent patients were entered at that level. Three patients were entered at each dose level and an interval of at least 4 weeks was required between the last patient entering a dose level and the next dose level commencing.

### Dose-limiting toxicity/maximum tolerated dose

Toxicities were graded according to the Common Toxicity Criteria, (CTC) version 2.0. Non haematological dose limiting toxicity (DLT) was defined as an episode of CTC grade 3 or 4 toxicity requiring dose modification. In the case of haematological toxicity, DLT was defined as an absolute neutrophil count of <1.0×10^9^ l^−1^ lasting for more than 7 days or associated with sepsis or an absolute platelet count of <50×10^9^ l^−1^ for more than 7 days or requiring platelet transfusion. Anaemia requiring transfusion was not classified as DLT. Any DLT occurring in two patients in a cohort resulted in the cohort being expanded from 3–6 patients. Dose escalation did not continue until no further episodes of DLT were observed in the expanded cohort.

The maximum tolerated dose was defined as the dose level at which DLT occurred in more than two thirds of patients enrolled in that level.

### Toxicity and response assessment

Blood was taken weekly for full blood count, differential, urea, creatinine, electrolytes and liver function tests. Treatment toxicity was assessed prior to each cycle. Left ventricular ejection fraction was measured by gated-pool radionuclide scan prior to cycle one and on completion of treatment. Audiometry was performed on entry into the trial and then as clinically indicated.

Tumour response was assessed by clinical examination, computed axial tomography (CT) and serum CA125 level. Clinical examination and CA125 were performed before each cycle and 4 weeks after the last cycle. CT scanning was performed prior to study entry, after every two courses and 4–6 weeks after the last treatment.

At each assessment, patients with progressive disease stopped treatment and those with stable disease or evidence of response received further cycles. The standard number of cycles was considered to be six; however treatment was continued for up to eight cycles if there was ongoing disease response. Interval cytoreductive surgery was permitted if clinically indicated.

### Dose modification

Patients who experienced haematological DLT received no further doxil. It was expected that the main DLT would be mucosal toxicity rather than myelosuppression. Thus, it was not planned that G-CSF should be routinely incorporated into the regimen. Those who developed any grade of palmar-plantar syndrome (PPS) were given pyridoxine 50 mg thrice daily. Grade 3 or 4 stomatitis or PPS resulted in a treatment delay of 1 week and then treatment continued with a 25% reduction in doxil dose, provided sufficient healing had occurred. Patients who had persistent grade 3 or 4 toxicity after 1 week off chemotherapy received no further doxil.

Mild-to-moderate doxil-related anaphylactoid reactions were managed by reducing the infusion rate and giving additional antihistamines and steroids as appropriate. Patients who had severe or life-threatening reactions received no further doxil. Anapylactoid reactions to doxil are not considered to be dose-related, so their occurrence was not classified as DLT. Patients who experienced such a reaction were taken off trial and replaced at that dose-level.

### Antitumour activity

Tumour response was assessed using standard WHO criteria. Complete response (CR) required normalisation of serum CA125 in addition to disappearance of all known disease. All responses had to be confirmed by two observations not less than 4 weeks apart. CA125 response was defined using the criteria described by Rustin and colleagues (Bridgewater *et al*, 1999).

## RESULTS

### Patient characteristics

Thirty-one patients were entered into the trial and their characteristics are summarised in Table 2

**Table 2:**
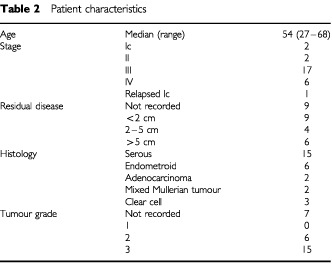
Patient characteristics

. Three patients had anaphylactoid reactions to the first dose of doxil. Thus 28 patients were evaluated for toxicity. Twenty-six patients had epithelial ovarian cancer and two patients had mixed Mullerian tumours of the ovary. The median age of the patients was 54 years and most had advanced disease; two patients had stage Ic and two had stage II disease, and one patient had relapsed ovarian cancer having originally had no chemotherapy for a stage I tumour.

### Toxicity

Nonhaematological toxicity at each level dose level is shown in Table 3

**Table 3:**
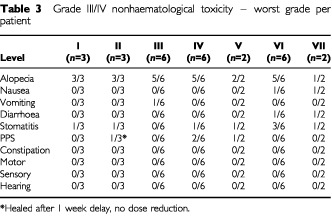
Grade III/IV nonhaematological toxicity – worst grade per patient

. At dose levels I, II and III grade 3/4 toxicity (excluding alopecia) occurred in four patients. Two patients had stomatitis (one each at level I and II), one patient had vomiting (level III) and one patient had short-lived grade 3 PPS (level II).

Increasing the doxil dose to 30 mg m^−2^ (level IV) resulted in grade 3/4 stomatitis (one patient), PPS (two patients) and infection (three patients, two episodes of neutropenic sepsis, one non-neutropenic). Two patients were enrolled at level V before it became apparent that DLT occurred at level IV. Both patients treated at level V suffered grade 3/4 side effects (stomatitis, PPS) and it was felt inappropriate to continue recruitment at this level. In view of the unacceptable toxicity resulting from 3-weekly liposomal doxorubicin, a protocol amendment, studying the effect of increasing the liposomal doxorubicin dose interval to 6 weeks (levels VI and VII) was submitted to and approved by both the institutional protocol review board and the institutional research ethics committee. At level VI, three patients had grade 3 stomatitis. One patient at this level developed grade 3 fatigue and declined further doxil. At level VII, one patient experienced grade 3 stomatitis and the other developed grade 3 stomatitis and grade 4 neurological toxicity (acute cerebellar ataxia) after cycle one. No cause was identified despite extensive investigation and she received no further doxil or paclitaxel.

No patients developed congestive cardiac failure or evidence of impaired left ventricular function on gated-pool scan. The mean change in ejection fraction for the 21 patients in whom two scans were available was −2% (range +13% to −16%).

CTC grade 3/4 haematologic toxicity for each dose level is shown in Table 4

**Table 4:**
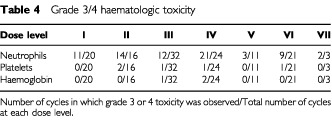
Grade 3/4 haematologic toxicity

. The majority of nadirs were short-lived. Haematologic DLT occurred in one patient out of the 12 treated at dose levels I–III (thrombocytopenia; grade 3, level III). Grade 3 thrombocytopenia occurred in an additional patient at this level, one cycle after the doxil was stopped due to stomatitis. At dose level IV there were two episodes of neutropenic sepsis with the neutropenia lasting 11 days in both cases. No haematological DLT was recorded in the two patients treated at dose level V. At level VI, one patient had neutropenic sepsis and two had prolonged neutropenia requiring cessation of liposomal doxorubicin. At level VII, only three courses which included the intended dose of doxil were given.

### Dose modifications

Table 5

**Table 5:**
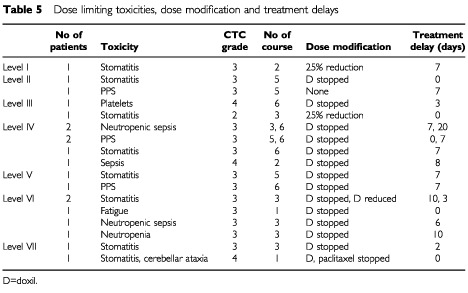
Dose limiting toxicities, dose modification and treatment delays

shows the DLTs, dose modifications and delays in treatment. The mean treatment delay per cycle was 0.5 days (level I, II), 0.3 days (level III), 2.3 days (level IV, V), 4 days (level VI). A dose reduction was made in one patient at dose level I (25% reduction in doxil) after cycle two because of grade 3 stomatitis. At level II, one patient had doxil stopped without treatment delay after four cycles for grade 3 stomatitis. At level III one patient had doxil stopped after five cycles for grade 4 thrombocytopenia requiring platelet transfusion. An additional patient in this level had an incorrect 25% dose reduction in doxil after cycle two for grade 2 stomatitis but received no further treatment due to disease progression. All patients treated at dose levels IV and V required dose modification during treatment. Five of six patients at level VI and both patients in level VII required dose modification.

Two patients died at dose level III, in both cases the deaths were not thought to be treatment-related. The first patient had been anticoagulated for a recently diagnosed proximal deep vein thrombosis. She collapsed suddenly 5 days after cycle one and a diagnosis of pulmonary embolism was made. The second patient died 15 days after cycle six and although a clinical diagnosis of pneumonia was made by the general practitioner, on further investigation the mode of death was more consistent with pulmonary embolism.

### Maximum tolerated dose

The maximum tolerated doses were defined as level IV (carboplatin AUC 5, paclitaxel 175 mg m^−2^, doxil 30 mg m^−2^ q 21), level V (carboplatin AUC 5, paclitaxel 135 mg m^−2^, doxil 30 mg m^−2^ q 21) and level VI (carboplatin AUC 5, paclitaxel 175 mg m^−2^ q 21, doxil 40 mg m^−2^ q 42). Two possible phase II doses have been defined, level II (carboplatin AUC 6, paclitaxel 175 mg m^−2^, liposomal doxorubicin 30 mg m^−2^ q 28) and level III (carboplatin AUC 5, paclitaxel 175 mg m^−2^, doxil 20 mg m^−2^ q 21). At level III there was only one episode of grade 3 toxicity, thrombocytopenia resulting in a dose delay of 3 days. Three more patients were treated at this dose level without further DLT.

### Tumour response

Response data was collected regardless of dose level and number of courses completed which included doxil. Antitumour effects were apparent in 67% of patients with radiologically measurable or evaluable disease. The CA125 response rate was 87%.

## DISCUSSION

Doxil has been tested in a number of cancers, some considered responsive to anthracyclines, others thought to be refractory. In addition to the trials in ovarian cancer, single agent liposomal doxorubicin has been tested in breast cancer, (Ranson *et al*, 1997) soft tissue sarcoma (Judson *et al*, 2001) head and neck cancer (Harrington *et al*, 2001) melanoma (Ellerhorst *et al*, 1999) renal cell carcinoma (Law *et al*, 1994) and pancreatic carcinoma (Schwartz and Casper, 1995) Doxil has been studied in combination with other agents including docetaxel, (Malik *et al*, 1998; Drinkard *et al*, 1999; Hirsch *et al*, 1999) paclitaxel, (Israel *et al*, 1998; Langley *et al*, 1998; Moore *et al*, 1998; Woll *et al*, 1999) cisplatin, (Klein *et al*, 1999) vinorelbine (Burstein *et al*, 1999; Gebbia *et al*, 1999; Jahanzeb *et al*, 1999) and gemcitabine (Rivera *et al*, 2001). There have been no trials of doxil-carboplatin published. Four trials of doxil-paclitaxel have defined a MTD for doxil of 30 mg m^−2^ every 3 weeks with doses of paclitaxel ranging from 150–200 mg m^−2^. In these studies, dose-limiting toxicities include stomatitis, PPS and myelosuppression. Three dose-finding studies of the related taxane, docetaxel showed similar dose-limiting toxicity.

The current study has defined the maximum tolerated dose of doxil in combination with carboplatin-paclitaxel as 30 mg m^−2^ when given on a 3-weekly schedule. Increasing the dose interval of doxil to 6 weeks did not allow the dose administered to be increased. A dose of 40 mg m^−2^ administered 6 weekly resulted in dose-limiting toxicity in four of six patients. Similar dose-limiting toxicity was observed in a trial of doxil 60 mg m^−2^ q 6 weekly and paclitaxel 175 mg m^−2^ q 3 weekly (Langley *et al*, 1998).

The current study was not designed to assess efficacy but the response rates are comparable with other first-line regimens. However, it should be noted that our recommended phase II/III dose for doxil in combination with carboplatin-paclitaxel is less than the dose used in phase II studies of single agent doxil in relapsed disease. Future strategies include the sequential administration of doxil with platinum-paclitaxel, either as a single agent or in combination with either carboplatin or paclitaxel, and trials of so-called sequential couplets are already underway.

In conclusion, this is the first study to define schedules for a triple combination of carboplatin-paclitaxel-doxil that can be taken forward into future phase II or III studies. The dose schedules are as follows; carboplatin AUC 6, paclitaxel 175 mg m^−2^, doxil 30 mg m^−2^ q 28 and carboplatin AUC 5, paclitaxel 175 mg m^−2^, doxil 20 mg m^−2^ q 21. At these doses, the combination is well tolerated and feasible to administer.
